# Comparing the Healthy Nose and Nasopharynx Microbiota Reveals Continuity As Well As Niche-Specificity

**DOI:** 10.3389/fmicb.2017.02372

**Published:** 2017-11-29

**Authors:** Ilke De Boeck, Stijn Wittouck, Sander Wuyts, Eline F. M. Oerlemans, Marianne F. L. van den Broek, Dieter Vandenheuvel, Olivier Vanderveken, Sarah Lebeer

**Affiliations:** ^1^Department of Bioscience Engineering, University of Antwerp, Antwerp, Belgium; ^2^Department of Translational Neurosciences, Faculty of Medicine and Health Sciences, University of Antwerp, Antwerp, Belgium; ^3^Department of ENT, Head and Neck Surgery, Antwerp University Hospital, Antwerp, Belgium

**Keywords:** microbiome, amplicon sequence variants, upper respiratory tract, bacterial community types, nasopharynx, next-generation sequencing

## Abstract

To improve our understanding of upper respiratory tract (URT) diseases and the underlying microbial pathogenesis, a better characterization of the healthy URT microbiome is crucial. In this first large-scale study, we obtained more insight in the URT microbiome of healthy adults. Hereto, we collected paired nasal and nasopharyngeal swabs from 100 healthy participants in a citizen-science project. High-throughput *16S rRNA* gene V4 amplicon sequencing was performed and samples were processed using the Divisive Amplicon Denoising Algorithm 2 (DADA2) algorithm. This allowed us to identify the bacterial richness and diversity of the samples in terms of amplicon sequence variants (ASVs), with special attention to intragenus variation. We found both niches to have a low overall species richness and uneven distribution. Moreover, based on hierarchical clustering, nasopharyngeal samples could be grouped into some bacterial community types at genus level, of which four were supported to some extent by prediction strength evaluation: one intermixed type with a higher bacterial diversity where *Staphylococcus*, *Corynebacterium*, and *Dolosigranulum* appeared main bacterial members in different relative abundances, and three types dominated by either *Moraxella*, *Streptococcus*, or *Fusobacterium*. Some of these bacterial community types such as *Streptococcus* and *Fusobacterium* were nasopharynx-specific and never occurred in the nose. No clear association between the nasopharyngeal bacterial profiles at genus level and the variables age, gender, blood type, season of sampling, or common respiratory allergies was found in this study population, except for smoking showing a positive association with *Corynebacterium* and *Staphylococcus*. Based on the fine-scale resolution of the ASVs, both known commensal and potential pathogenic bacteria were found within several genera – particularly in *Streptococcus* and *Moraxella* – in our healthy study population. Of interest, the nasopharynx hosted more potential pathogenic species than the nose. To our knowledge, this is the first large-scale study using the DADA2 algorithm to investigate the microbiota in the “healthy” adult nose and nasopharynx. These results contribute to a better understanding of the composition and diversity of the healthy microbiome in the URT and the differences between these important URT niches.

**Trial Registration:** Ethical Committee of Antwerp University Hospital, B300201524257, registered 23 March 2015, ClinicalTrials.gov Identifier: NCT02 933983.

## Introduction

Respiratory tract infections, including acute and chronic otitis media in children and chronic rhinosinusitis in adults, are the most commonly treated health issues in primary care (Francis et al., 2009). The respiratory tract can be divided in the lower and upper respiratory tract (URT), where the latter comprises the anterior nares, the nasal passages, the paranasal sinuses, the naso- and oropharynx, and finally the larynx above the vocal cords (reviewed in Man et al., 2017). In Europe, URT infections account for 57% of all prescribed antibiotics, having a significant impact on the emerging problem of antibiotic resistance (van der Velden et al., 2013). Without evidence for a clear causative role for specific bacterial species, culture-based sampling has linked various opportunistic pathogens to chronic rhinosinusitis and otitis media. However, these bacterial species seem also to be present in healthy individuals (Lemon, 2010; Stearns et al., 2015). To better elucidate the contribution of the microbiota to URT diseases and to design targeted (anti-)microbial approaches, a better understanding of the composition and diversity of the “healthy URT microbiota" is essential.

Due to major advances in sequencing techniques and large-scale sequencing projects such as the NIH Human Microbiome Project, our understanding of the composition and functional properties of the human microbiota has improved greatly (Turnbaugh et al., 2007). While many studies have previously focused on the microbiota of the gastrointestinal tract, in recent years, interest in the resident microbial communities of other human body niches, such as the respiratory tract, has clearly been expanding.

The nose and nasopharynx are key niches of the URT. Both niches host commensals and potential pathogenic species that may cause airway infections under certain conditions (reviewed in Man et al., 2017). While the nasal microbial community is being mapped in large initiatives like the NIH Human Microbiome Project (Turnbaugh et al., 2007) and other more recent studies (Bassis et al., 2014; Camarinha-Silva et al., 2014; Biswas et al., 2015), the nasopharynx is less explored. Some larger studies (including 50 up to 234 participants) have profiled the nasopharyngeal microbiota in children with next-generation sequencing (NGS) (Bogaert et al., 2011; Biesbroek et al., 2014; Stearns et al., 2015; Teo et al., 2015; Bosch et al., 2017; Chonmaitree et al., 2017), but only a few studies have investigated the healthy adult nasopharyngeal microbiota (Ling et al., 2013; Cremers et al., 2014; Stearns et al., 2015). These studies in adults were small-scale (including less than 40 participants), with different age groups and populations from different geographical locations. Furthermore, with the exception of the study of Stearns et al. (2015), these studies used *16S rRNA* gene pyrosequencing, which has some limitations over the more in-depth Illumina MiSeq sequencing. Main limitations are sequencing errors in homopolymeric regions and lower sequencing depth. In addition, all these studies have used a clustering approach where sequences were clustered into operational taxonomic units (OTUs). While this approach is most often used, it currently underutilizes the power of high-quality sequences produced by modern sequencing technologies, such as Illumina MiSeq (reviewed in Hugerth and Andersson, 2017). Therefore, alternative algorithms that detect more fine-scale variations like MED (Eren et al., 2014, 2016) and Divisive Amplicon Denoising Algorithm 2 (DADA2) (Callahan et al., 2016) have recently emerged, resulting in improved precision of diversity and dissimilarity measures. Since both the nose and the nasopharynx are low-complexity (in terms of observed richness or total number of bacterial genera present) and low-biomass (in terms of total amount of bacterial cells) microbial niches (Biesbroek et al., 2012), an accurate discrimination between these biological variants is essential. The recently developed DADA2 algorithm in combination with Illumina MiSeq sequencing has the potential to improve sensitivity, specificity, and reproducibility compared to OTU-picking methods. This algorithm infers unique biological variants called “amplicon sequence variants” or ASVs (Callahan et al., 2017) by correcting sequencing errors in the reads. The ASV concept is an alternative to the classical concept of an OTU: OTU-based strategies perform clustering based on a fixed percentage identity threshold (e.g., 97%), while ASVs are the result of a denoising procedure only. The DADA2 denoising strategy is based on the quality scores of all reads as well as the abundance distribution of the unique sequences.

By the implementation of this DADA2 pipeline, we explored here the diversity and main bacterial members of the nose and nasopharynx of 100 healthy participants in order to obtain more insight in the commensal and potential pathogenic bacteria colonizing these URT niches. The resulting bacterial profiles were mined for associations to the data available from our healthy volunteers, such as age, gender, blood type, smoking, season, and blood analyses for total immunoglobulin E (IgE) and IgE levels against common respiratory allergies.

## Materials and Methods

### Study Design and Sample Collection

Participants between 18 and 65 years old without acute or chronic URT diseases were recruited between July 2015 and October 2016 via the University of Antwerp and a Belgian–Dutch citizen-science platform^1^, after approval of the study by the Ethical Committee of the Antwerp University Hospital/University of Antwerp (registration number B300201524257, registered 23 March 2015, ClinicalTrials.gov Identifier: NCT02933983). A written informed consent was obtained from all participants, as well as a blood sample and a questionnaire with general information on their medical history and additional information such as smoking behavior. Participants who received antibiotics (self-reported) in the past year or suffered from acute or chronic airway infections were excluded from the study. In total, 90 nasal and 100 nasopharynx samples were collected in a standardized way by the responsible ear, nose, and throat (ENT) specialist with flocked swabs (Copan, 503CS01) at the level of the anterior nasal cavity, and nasopharynx. All samples were immediately suspended in 750 μl MoBio bead solution (PowerFecal^®^ DNA Isolation Kit; MO BIO Laboratories Inc., Carlsbad, CA, United States) and placed on ice prior to DNA extraction. DNA extraction took place within 4 h after sample collection. DNA samples were stored at -20°C until further use.

### Blood Analysis for Total and Specific IgE

A serum sample was collected from all participants in order to investigate the total IgE level in their blood, as well as some specific IgEs for respiratory allergies (tree pollen, grass pollen, and house dust mite). Blood samples were collected at the Antwerp University Hospital by a responsible nurse. Total and specific IgEs were quantified by an ImmunoCAP System (Thermo Fisher Scientific, Uppsala, Sweden). All assays were performed and results interpreted according to the manufacturers’ recommendation. Total IgE counts below 114 kU/l were considered as non-allergic. For specific IgE counts, values below 0.35 kUA/l were considered as non-allergic.

### DNA Extraction

The PowerFecal^®^ DNA Isolation Kit (with Inhibitor Removal Technology^®^) was used according to the instructions of the manufacturer. DNA concentrations were measured with a Qubit^®^ 3.0 Fluorometer (Life Technologies, Ledeberg, Belgium). DNA extractions were performed in a laboratory room dedicated for DNA/RNA extraction, physically separated from the microbiology room to minimize contamination.

### Illumina MiSeq *16S rRNA* Gene Amplicon Sequencing

The primers used for Illumina MiSeq sequencing were based on the previously described 515F-806R primers (Caporaso et al., 2010) and altered for dual-index paired-end sequencing, as earlier described (Kozich et al., 2013). Briefly, each DNA sample was subjected to dual barcoded PCR, amplifying the V4 region of the *16S rRNA* gene using Phusion High-Fidelity DNA polymerase (New England Biolabs, United States). PCR products were purified by the Agencourt AMPure XP Magnetic Bead Capture Kit (Beckman Coulter, Suarlee, Belgium), and quantified using the Qubit^®^ 3.0 Fluorometer. The library was prepared by pooling all PCR samples in equimolar concentration and loaded onto a 0.8% agarose gel to remove remaining primer dimers from the product. The product was purified by gel extraction using the NucleoSpin^®^ Gel and PCR clean-up (Macherey-Nagel). The final library concentration was determined with the Qubit^®^ 3.0 Fluorometer. The library was denatured with 0.2 N NaOH (Illumina), diluted to 7 pM and spiked with 10% PhiX control DNA (Illumina). The library was loaded onto the flow cell of the v2 Chemistry MiSeq Reagent Kit (paired-end dual indexing sequencing; 2 × 250 bp kit; Illumina, San Diego, CA, United States) on the MiSeq Desktop Sequencer (M00984, Illumina) at the Centre of Medical Genetics, University of Antwerp, Belgium. The sequencing data were deposited in ENA under accession number PRJEB23057.

### Sequence Processing and Quality Control

Processing and quality control of reads was performed using the R package dada2, version 1.4.0 (Callahan et al., 2016). After inspection of quality control profiles, the first 35 bases of all reverse reads were trimmed since they frequently contained uncalled bases. Next, all reads containing remaining uncalled bases or more than three expected errors were removed. Afterward, the parameters of the DADA2 error model were learned from a random subset of 1 million reads. This error model was then used to denoise all sequences; i.e., to infer the ASVs. Denoised reads (ASVs) were then merged and read pairs with one or more conflicting bases between the forward and reverse read were removed. Chimeric sequences were then detected and removed using the function “removeBimeraDenovo.” Finally, reads (ASVs) were classified from the kingdom to the genus level using the Silva reference *16S rRNA* gene database, version 123 resulting in the construction of an ASV table with read counts of all ASVs in all samples.

In the next phase, quality control was performed on the level of the ASVs and samples. ASVs longer than 251 bases were removed, as well as ASVs classified as Archaea, chloroplasts, or mitochondria. The PCR and DNA extraction negative controls were inspected, and ASVs classified as known contaminants and/or that were overrepresented in the negative blank controls (when compared to the samples) were removed. Finally, samples were subjected to quality control based on total read count and read count per sample volume pooled. Samples were required to contain at least five times more reads per volume than the negative controls, as well as more than 1000 total reads.

### Biostatistical Analysis

Processing of the ASV table, ASV annotations (e.g., classification), and sample annotations (metadata) were performed using the in-house R package “tidyamplicons,” publicly available at github.com/SWittouck/tidyamplicons. For the analyses on the genus level, ASV read counts were aggregated on the genus level or, if unavailable, on the most specific level at which taxonomic annotation was available. Alpha-diversity was explored at the genus level using two different metrics: the number of observed genera and the inverse Simpson metric (defined as the inverse probability that two random reads belong to the same taxon). Differences in these two metrics between the nose and nasopharynx samples were tested using a Wilcoxon rank-sum test. Correlation of the alpha-diversity metrics between the nose and nasopharynx was assessed using Pearson correlation and the corresponding significance test implemented in the cor.test function in R. For beta-diversity analysis, the Bray–Curtis distance was used, defined as the summed differences in read counts for all taxa, divided by the total read counts in both samples. The Bray–Curtis beta-diversity matrix was explored visually using principal coordinates analysis (PCoA). In order to test the bacterial profiles for clustering structure, we made use of the prediction strength metric (Tibshirani and Walther, 2005). First, all samples (from both nose and nasopharynx) were clustered in seven clusters using hierarchical clustering with the unweighted pair group method with arithmetic mean (UPGMA) on the Bray–Curtis distance matrix. Clusters containing only one or two samples were considered outliers and were removed, since those cannot be evaluated using prediction strength. Next, four distance matrices were calculated: Bray–Curtis (on relative abundances, as usual), Bray–Curtis on the presence/absence level, Jensen–Shannon divergence, and Jensen–Shannon distance (equal to the square root of the Jensen–Shannon divergence). Two clustering techniques were then performed on those matrices: UPGMA and partitioning around medoids (PAM). Each distance metric – clustering algorithm combination was performed for different numbers of clusters (2 up until 10). This approach to evaluate clustering largely follows Koren et al. (2013), except that we added the UPGMA clustering method and the presence/absence Bray–Curtis distance metric.

The association of the nasopharyngeal microbiota with participant metadata was performed for all metadata variables that had more than six participants in at least two categories. These variables were gender, age, blood type, smoking, season of sampling, total IgE level, and specific IgE levels for house dust mite, grass pollen, and tree pollen. For each of these variables, the association with the microbiota was tested using a permanova test implemented in the function “adonis” of the R package “vegan.” Specifically, the adonis function tests whether the Bray–Curtis distances within groups of samples are smaller than the distances between groups; significance is assessed using a permutation strategy. The association between the variable “smoking” and the genera *Corynebacterium*, *Dolosigranulum*, and *Staphylococcus* was tested with a Wilcoxon rank-sum test.

For the analyses on the ASV level, only samples were retained of participants that had both a nose and a nasopharynx sample passing quality control. To test whether ASV presence was correlated between both niches, the following strategy was used for each ASV. First, a two-way frequency table was constructed, where each cell contained a count of participants and the variables were “presence of the ASV in the nose” and “presence of the ASV in the nasopharynx.” Next, association between the two variables was tested using a Fisher’s exact test (implemented in the base R function fisher.test). All ASVs were then visualized in a scatterplot with on the *x*-axis the expected proportion of co-occurrence under the assumption of no correlation and on the *y*-axis the observed proportion of co-occurrence. The expected proportion of co-occurrence was calculated by multiplying the occurrence proportion in the nasopharynx with the occurrence proportion in the nose. To test preference of ASVs for one of the niches of the other, a similar approach was followed. First, a two-way frequency table was constructed where each cell contained a count of samples and the variables were “sample type (nose or nasopharynx)” and “presence/absence.” Preference for the nose or nasopharynx was then assessed by testing the association between these two variables using a Fisher’s exact test. Finally, the ASVs were visualized in a scatterplot with on the *x*-axis the occurrence proportion in the nose and on the *y*-axis the occurrence proportion in the nasopharynx.

Quality control, biostatistical analysis, and visualization were performed in R version 3.4.1. All visualizations were created using ggplot2 version 2.2.1 (Wickham, 2009). Vegan version 2.4.3 (Oksanen et al., 2016) was used for alpha- and beta-diversity analyses.

## Results

### The Adult Nasopharynx Is Dominated by at Least Four Bacterial Community Types at the Genus Level

Samples from the “healthy” nose and nasopharynx of participants with no signs of URT infections, recruited in collaboration with a Belgian–Dutch citizen-science platform, were collected. In total, we collected 90 nasal samples and 100 nasopharyngeal samples, of which, respectively, 84 and 92 remained after quality control. Supplementary Table S1 presents the different steps in the quality control with the remaining amount of reads after each step.

**FIGURE 1:**
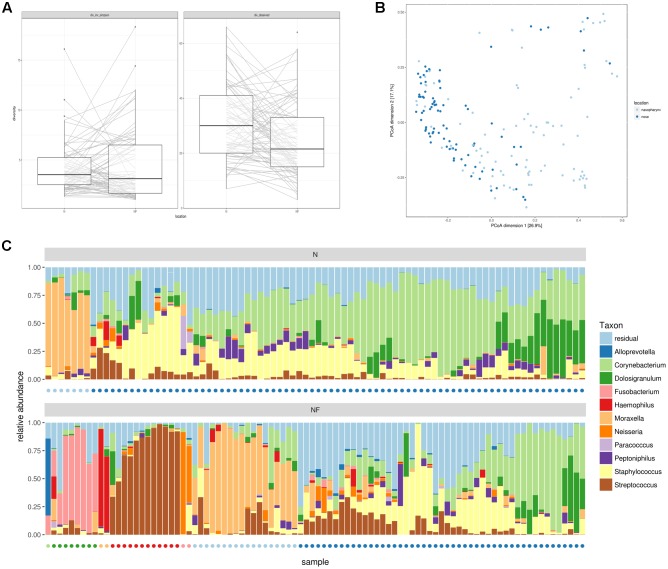
Alpha and beta-diversity measures for nasal (*n* = 84) and nasopharynx (*n* = 92) samples from healthy adults without acute or chronic airway infections. **(A)** Inverse Simpson index and observed richness for nasal and nasopharynx samples at genus level. **(B)** PCoA to compare bacterial taxonomy between samples at the same location and inter-individual variation between locations. **(C)** Hierarchical clustering at genus level of nose (Top) and nasopharynx (Bottom) samples. Most nose samples have an intermixed profile, where *Staphylococcus*, *Corynebacterium*, and/or *Dolosigranulum* appear to be key members. A subset of nose samples is *Moraxella*-dominated. Nasopharynx samples show seven bacterial community types in the nasopharynx depending on the dominant genera: *Moraxella-*, *Haemophilus-*, *Neisseria-*, *Alloprevotella-*, *Streptococcus-*, or *Fusobacterium-*dominated, or the more diverse and intermixed type with *Staphylococcus*, *Corynebacterium*, and/or *Dolosigranulum* as important members. Colored dots indicate the cluster assignment of the samples.

Alpha and beta-diversity measures of both nose and nasopharynx swabs were calculated to estimate the bacterial *16S rRNA* gene diversity in these adult URT niches. **Figure 1A** presents the overall observed richness and the inverse Simpson index in each participant for both the nose and the nasopharynx. Both niches contain a rather low number of observed genera (on average 31 in the nose and 25 in the nasopharynx) with a significantly higher observed richness in the nose than in the nasopharynx (*p* = 0.002). Additionally, the low inverse Simpson index (on average 4.1 for the nose and 4.3 for the nasopharynx) suggests an uneven distribution of the abundance of this limited amount of genera, indicating that both the adult nose and the adult nasopharynx are low-diversity niches where only a limited number of bacterial genera are dominant. Finally, the correlation of alpha-diversities between the nose and nasopharynx was calculated to be 0.19 and 0.21 for observed richness and inverse Simpson, respectively, meaning that the amount of genera in the nose is only weakly informative for the amount of genera in the nasopharynx and vice versa.

Next, we investigated the microbial composition of all samples and explored differences between these major URT niches as well as interpersonal variation. PCoA was first used to visualize this variation (**Figure 1B**). Nose and nasopharynx samples appeared mostly intermixed, except for one group of samples consisting of exclusively nasopharynx samples. Then, we visualized the genera with the highest overall abundance in the nose and nasopharynx and performed hierarchical clustering (**Figure 1C**). Based on this clustering, we observed seven potential “community types” in the nasopharynx, with different taxonomic composition. A dendrogram of the hierarchical clustering can be found in Supplementary Figure S1. Almost half of the participants showed a clear dominance of one of the following genera: *Moraxella* (19.6%), *Streptococcus* (13%), *Fusobacterium* (8.7%), *Neisseria* (2.2%), *Alloprevotella* (1.1%), or *Haemophilus* (2.2%). The other nasopharynx samples (53.3%) contained an intermixed bacterial profile where *Staphylococcus*, *Corynebacterium*, and *Dolosigranulum* seemed to be important bacterial members with varying relative abundances. In the nose, the inter-individual variation at genus level was smaller. Only two community types could be observed in the nose, with most samples (91%) showing the intermixed diverse profile and a smaller number of samples (9%) the *Moraxella*-dominated profile. To evaluate the significance of the observed clusters, the prediction strength was calculated for a varying number of clusters, using Bray–Curtis as well as three alternative distance metrics (Supplementary Figure S2, left). For up to four clusters (i.e., *Streptococcus*, *Fusobacterium*, *Moraxella*, and the intermixed type) obtained using hierarchical clustering, strong to moderate support was observed based on this prediction strength. Supplementary Figure S3 shows the PCoA plot with indication of these four clusters. The significance of the three smaller clusters (*Neisseria*, *Haemophilus*, and *Alloprevotella*) should be further confirmed in larger study groups. For clusters obtained using PAM, little to no support was found (Supplementary Figure S2, right). We believe therefore that the observed community types should not be seen as discrete clusters but rather as a continuum, where participants can belong to a given type or be situated in-between multiple types.

### The Nasopharyngeal Bacterial “Community Types” Show an Association with Smoking Behavior, But Not with Gender, Age, Blood Type, Season of Sampling, and Common Respiratory Allergies

**FIGURE 2:**
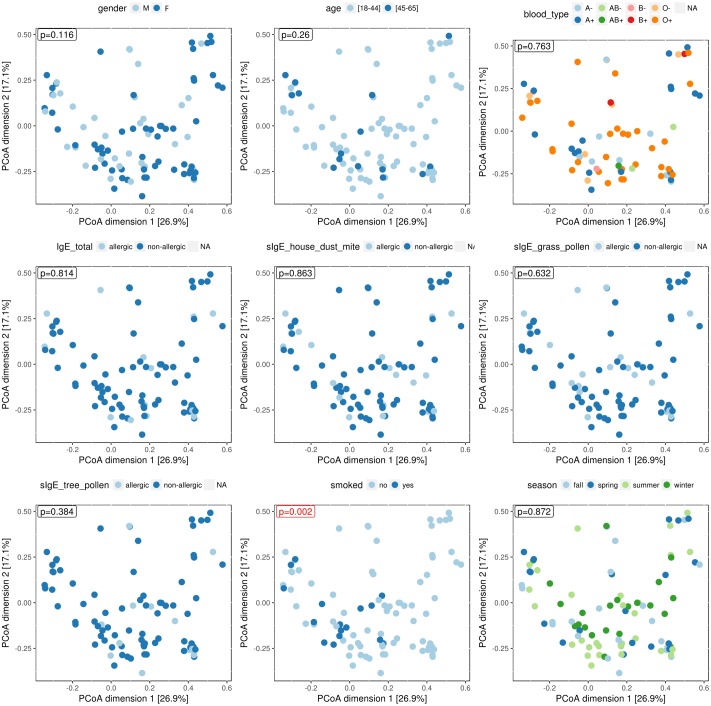
Principal coordinates analysis (PCoA) plots to visualize potential correlations with several host- and environment-related variables. Age, gender, blood type, smoking, season of sampling, total IgE, and specific IgEs for tree pollen, grass pollen, and house dust mite were investigated in relation to the bacterial profiles in the nasopharynx. *P*-values – based on permanova – are shown for all tested covariates and colored red when statistically different (here only for smokers) (*p* = 0.002).

We recorded several variables for each participating volunteer: age, gender, blood type, smoking and season (sampling date) to investigate possible associations with the nasopharyngeal microbial profiles (Supplementary Table S2). Each of the variables was visualized on a PCoA plot to look for potential associations with the bacterial composition of the samples, using permanova for statistical analysis (**Figure 2**). We divided our study population (mean age = 34.78, *SD* = 11.2, range = 18–65) in two age categories, 18–45 years (84% of the study population) and 45–65 years (16%) but could not demonstrate an association with these age classes and the bacterial profiles in our – quite young – study population. Also gender (34 males and 58 females), blood type, and season were not found to be associated with the nasopharyngeal microbiota. Smoking behavior, however, showed an association with the bacterial profiles in the nasopharynx (*p* = 0.002). Participants who smoke or used to smoke (17% of the study population) seemed to almost all have an intermixed bacterial profile with high relative abundances of *Staphylococcus*, *Corynebacterium*, and *Dolosigranulum*, with the exception of one participant in the *Haemophilus*-dominated group. Because smoking behavior showed a positive association with the intermixed nasopharyngeal “community type,” we investigated this association further at genus level. We found a positive association of smoking with *Corynebacterium* (Wilcoxon rank-sum test, *p* = 0.002) and *Staphylococcus* (*p* = 0.02), while *Dolosigranulum* showed no association (Supplementary Figure S4).

In addition to these more descriptive variables, a blood sample of each participant was analyzed for total blood IgE and some IgEs specific for respiratory allergens, such as house dust mite, grass pollen, and tree pollen (Supplementary Table S1). Subjects with total IgE and specific IgE counts above 114 kU/l and 0.35 kUA/l, respectively, were considered as “allergic.” In our study population, 15% of our participants was considered allergic based on total IgE and 25, 25, and 14% were allergic for house dust mite, grass pollen, and tree pollen, respectively. For all participants, no clear association was found with the tested respiratory allergies and the genus-abundance profiles of their nasopharyngeal microbiota.

### Intragenus-Information on Co-occurrence of ASVs in the Nose and Nasopharynx

**FIGURE 3:**
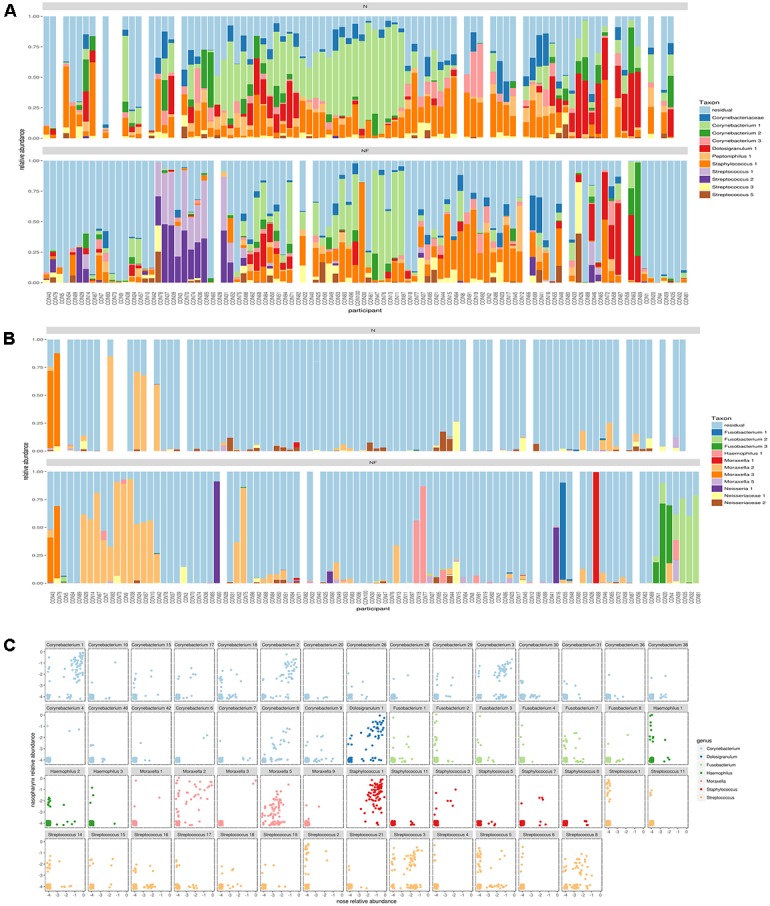
Comparison of amplicon sequence variants (ASVs) in nose and nasopharynx. **(A)** ASVs of Gram-positives in paired samples of the nose (Top) and nasopharynx (Bottom). White bars indicate samples that were left-out during the processing. **(B)** ASVs of Gram-negatives in paired samples of the nose (Top) and nasopharynx (bottom). White bars indicate samples that were left-out during processing. **(C)** Combined plot of nose and nasopharynx for the different ASVs belonging to the dominant genera of the observed community types in this study. Only ASVs occurring in at least three samples were visualized.

In order to be able to distinguish different variants within one genus, we applied the DADA2 pipeline and found for many of the abundant genera multiple ASVs in both nose and nasopharynx. ASVs were then aligned to the SILVA database (version123) to get an overview of all species with V4 sequences identical to the ASV. This gives a general idea of the classification of the ASV at the sub-genus level. In order to be able to visualize more ASVs, we divided Gram-positive (**Figure 3A**) and Gram-negative (**Figure 3B**) ASVs. For *Corynebacterium*, the most abundant ASVs found in the nose could be classified as *Corynebacterium accolens/macginleyi* (Corynebacterium1) and *Corynebacterium propinquum/pseudodiphtheriticum* (Corynebacterium2). Interestingly, these variants were also present in the nasopharynx, although less frequent (**Figure 3A**). For *Moraxella*, three abundant variants were detected in both the nose and nasopharynx: *Moraxella porci* (Moraxella1), *Moraxella catarrhalis/nonliquefaciens* (Moraxella2), and *Moraxella bovoculi/lacunata/equi* (Moraxella3). The Moraxella2 variant, *M. catarrhalis/nonliquefaciens*, was most abundant in different samples and almost never co-occurred with the other Moraxella variants in the same sample. Of interest, the two persons hosting the third *Moraxella* variant in the nasopharynx also had this variant in their nose. Furthermore, some *Streptococcus* ASVs could be discriminated in the nasopharynx of which Streptococcus1 was further classified as *Streptococcus pneumoniae/pseudopneumoniae* and Streptococcus3 as *Streptococcus dentisani/tigurinus/oralis/oligofermentans/mitis/infantis/gordonii*. Interestingly, the latter variant was present in 1–15% abundance in a large part of our nasopharynx samples of the healthy adults, while the ASV classified as *S. pneumoniae/pseudopneumoniae*, which is described in literature as a common URT pathogen, dominated the samples if present. The Streptococcus2 ASV was found to co-occur with Streptococcus1 and in addition, their sequences differed in only one nucleotide. Therefore, it is likely that they originate from two different copies of the *16S rRNA* gene of the same strain. Only one abundant *Haemophilus* ASV was found (*Haemophilus haemolyticus/influenzae*) in the nasopharynx. For *Dolosigranulum*, also only one abundant ASV was identified, which we could classify *as D. pigrum*, and this ASV seemed to be more abundant in the nose than the nasopharynx. Finally, three different *Fusobacterium* variants were observed in the nasopharynx samples (Fusobacterium1; *Fusobacterium nucleatum/canifenilum*, Fusobacterium2; *F. nucleatum*, and Fusobacterium3; *F. nucleatum/naviforme*). In addition to the most abundant ASVs discussed above (**Figures 3A,B**), other ASVs showing a lower abundance within the genera of the “community types” were detected, of which some were only present in the nasopharynx, such as Haemophilus1 and 3, Fusobacterium2, and Streptococcus1,4, and 6 (**Figure 3C**). Supplementary Figures S5a–h give a more detailed comparison of paired nose and nasopharynx samples at ASV level for each of the dominant genera of the bacterial community types, showing the unique niche-specificity of some ASVs, while other ASVs are less niche-related and show continuity between both niches. The co-occurrence and niche-specificity for other ASVs was also visualized and was statistically tested using a Fisher’s exact test (Supplementary Figures S6, S7). For example, Streptococcus1 and Fusobacterium2 only appeared in nasopharynx samples studied, while Corynebacterium2 and Moraxella2 occurred in both niches. It should be noted that ASVs presented here are determined based on the amount of variation present in the V4 region of the *16S rRNA* gene. This implicates that absence of detection of different ASVs within a genus does not mean that they are not present. Therefore, for example, a distinction between different *Staphylococcus* species, such as the potential pathogenic *S. aureus* and more commensal *S. epidermidis*, was not possible here.

## Discussion

Upper respiratory tract infections have a major impact on public health. Insight into the bacterial communities colonizing different URT niches might help to better understand the role of bacteria in the URT in health and disease. Here, microbial DNA from paired nasal and nasopharynx samples of 100 healthy adult participants recruited through a citizen-science project was isolated and sequenced. After rigorous quality control of these samples, 84 nose and 92 nasopharynx samples remained for further analysis of microbial diversity and dominant taxa present in these niches.

Contrary to previous studies using *16S rRNA* gene pyrosequencing to investigate bacterial communities present in the nose and nasopharynx (Ling et al., 2013; Biesbroek et al., 2014; Cremers et al., 2014), we applied Illumina MiSeq sequencing technology – combined with analysis up to unique ASV level (Callahan et al., 2016) – to capture the entire bacterial diversity in the relatively low-biomass niches of the nose and nasopharynx. The mean observed richness was significantly higher in the nose (31 genera), compared to the nasopharynx (25 genera). Although we did not use classical OTU clustering, our observations are in line with previous work, showing a low observed richness in the nasopharynx (Cremers et al., 2014; Stearns et al., 2015). The fact that only a limited number of bacterial genera are able to colonize the nose and nasopharynx – as shown by the low inverse Simpson index – indicates that these niches have specific challenges to which colonizing bacteria need to be adapted. Since the nose has a continuous inflow of air and dust particles (open ecosystem), it is not surprising that it is colonized by a more diverse range of bacteria compared to the nasopharynx. Together with some dominant bacterial species, the nose is also colonized with a number of low-abundant taxa that might originate from the air, the skin, or other external sources, as suggested in source-tracking studies, such as done by Lax et al. (2014). The nasopharynx, on the other hand, can be seen as a cavity or microbial bioreactor, which is more isolated from the external environment and for which the host forms a more selective environment. These unique features in both ecosystems possibly lead to niche-specific factors that are involved in colonization (reviewed in Man et al., 2017). In our data, we could observe, at the level of ASVs, that some *Haemophilus*, *Fusobacterium*, and *Streptococcus* ASVs are mainly present in the nasopharynx. This might be caused because these taxa are better adapted to the nasopharynx conditions such as its stratified squamous epithelium, higher temperature, and lower pH (reviewed in Man et al., 2017) (Supplementary Figure S7). We also observed that certain ASVs always co-occurred in both the nose and the nasopharynx (e.g., several *Moraxella* and *Corynebacterium* ASVs) (Supplementary Figure S6). Thus, we found that ecological processes, such as dispersal (e.g., movement of organisms across spaces) and selection have an important impact on the bacterial communities that reside in the URT, as also nicely reviewed in de Steenhuijsen Piters et al. (2015).

In addition, we also aimed to explore the dominance of bacterial taxa and the possible occurrence of community types, similarly as previously done in the gastrointestinal tract (referred to as “enterotypes”) (Arumugam et al., 2011), the vaginal tract (reviewed in Petrova et al., 2015), and the nasopharynx of newborns (Biesbroek et al., 2014). Based on hierarchical clustering, our samples could be grouped by the dominant genera, resulting in at least four bacterial “community types” for the nasopharynx samples analyzed here: *Moraxella-*, *Fusobacterium-*, or *Streptococcus-*dominated, or one more intermixed type showing a higher bacterial diversity where *Corynebacterium*, *Staphylococcus*, and/or *Dolosigranulum* are the main members of the bacterial community. Some smaller clusters (*Haemophilus*, *Alloprevotella*, and *Neisseria*) were also observed, but their significance should be confirmed in larger studies. Since some (albeit exceptional) samples show a mixture of genera from different clusters, these profiles should not be interpreted as discrete community types but rather as a continuum. In contrast, nose samples mainly showed the diverse type with again *Corynebacterium*, *Staphylococcus*, and *Dolosigranulum* as the main members. This last observation is in agreement with previous studies demonstrating that these genera are highly abundant in the anterior nares (Bassis et al., 2014; Camarinha-Silva et al., 2014; Biswas et al., 2015). A minority of the nose samples (9%) was dominated by *Moraxella.* Of note, the biological relevance of such discrete community types in other human body niches, such as the vagina and gastrointestinal tract, is still under debate and needs further substantiation (Koren et al., 2013). This will certainly also be the case for the nasopharynx, for which our present study should merely be seen as a starting point, since the clusters observed in this study were only confirmed to some extent for hierarchical clustering on a Bray–Curtis distance metric.

The nasopharyngeal microbiota in healthy adults was – to the best of our knowledge – not yet investigated in large study cohorts. However, Biesbroek et al. (2014) demonstrated the bacterial succession of the nasopharynx microbiota in Dutch children. They found the infant nasopharynx to be mostly dominated by *Moraxella*, *Haemophilus*, or *Streptococcus*. Interestingly, our data show that these three genera are also maintained in the nasopharynx of adults, both in the age class 18–45 and 45–65 years. In young children, a *Streptococcus*-dominated bacterial profile was found to be associated with a less-stable nasopharyngeal microbiota, thereby potentially increasing the risk of URT infections (Biesbroek et al., 2014). The same study suggested that *Moraxella-* or *Dolosigranulum*/*Corynebacterium*-dominated bacterial profiles might be beneficial for respiratory health, which was also suggested before (Laufer et al., 2011). In contrast, Santee et al. (2016) found the enrichment of *Moraxella* in the nasopharynx of children, in particular *Moraxella nonliquefaciens*, to be associated with acute sinusitis.

The differences observed between studies such as by Biesbroek et al. (2014) and Santee et al. (2016) might be caused by the difference in molecular techniques used (e.g., phylogenetic microarray vs. pyrosequencing, respectively) and by the limitation of sequencing techniques to distinguish between discrete species within a genus. Therefore, we used the recently described DADA2 pipeline that is able to distinguish sequence variants differing by as little as one nucleotide (Callahan et al., 2016) to investigate intragenus variation, described as unique ASVs. We could further classify the most abundant *Moraxella* and *Streptococcus* ASVs in our healthy study group as *Moraxella nonliquefaciens/catarrhalis* and *S. pneumoniae/pseudopneumoniae*, two species well documented in the literature to have potential as URT pathogens (de Vries et al., 2009; Goldstein et al., 2009; van der Poll and Opal, 2009). Future studies need to investigate whether the presence and abundance of these ASVs is linked to susceptibility for airway diseases, such as chronic rhinosinusitis. In general, potential pathogenic ASVs such as *S. pneumoniae/pseudopneumoniae* (van der Poll and Opal, 2009) and *H. haemolyticus/influenzae* (Duell et al., 2016) were more present in the nasopharynx compared to the nose. On the other hand, some of the ASVs that we found, such as *Streptococcus*3 (with hits in the SILVA database to among others *S. oralis* and *S. mitis*) and *Dolosigranulum*, have shown potential as probiotics for the URT in other studies (Roos et al., 2001; Tano et al., 2002; Laufer et al., 2011; Biesbroek et al., 2014).

The possible existence of nasopharyngeal community types raises the question which host and environmental factors are associated with these community types. Several available variables obtained from our healthy study population were analyzed here, including age, gender, blood type, smoking, common respiratory allergies, and season. We could not observe an association between our tested variables and the microbiota, except for the variable smoking, where we observed a positive correlation between smoking and the nasopharyngeal dominant genera (*p* = 0.002). The nasopharyngeal microbiota of smokers or ex-smokers appeared to be associated with the genus *Corynebacterium* (*p* = 0.002) and *Staphylococcus* (*p* = 0.02). Although some studies suggest a possible link between cigarette smoke and the URT microbiota (Charlson et al., 2010; Yu et al., 2017), this link still remains unclear and needs further research. We should note, however, that the identification of microbiome-associated variables is extremely challenging and large study-cohorts are probably necessary to identify such associations, as nicely shown for the gut microbiome (Falcony et al., 2016).

## Conclusion

Our findings indicate that the healthy adult nasopharynx can be grouped into some bacterial community types, each dominated by different genera. For up to four clusters, their significance was supported with prediction strength evaluation: *Moraxella*-dominated, *Streptococcus-*dominated, *Fusobacterium*-dominated, or a more intermixed diverse type where *Corynebacterium*, *Staphylococcus*, and/or *Dolosigranulum* appeared to be key bacterial members. Of these types, some were highly nasopharynx-specific, and never dominant in the nose, for instance the *Fusobacterium*- and *Streptococcus*-dominated type. By using the DADA2 pipeline, we could observe intragenus variation in the nose and nasopharynx and found both commensal as well as potential pathogenic bacteria present in the “healthy” URT. Several variables that could possibly influence these bacterial profiles were investigated, but a positive association could only be found between smoking and the occurrence of *Corynebacterium* and *Staphylococcus* in our study population. Future studies should be performed to determine how stable these bacterial profiles are and whether they are associated with susceptibility to the development of URT diseases.

## Author Contributions

SL conceived the study. IDB, StW, DV, OV, and SL designed different aspects of the study protocols. OV was the responsible ENT specialist. Laboratory work was performed by IDB and EO. Bioinformatic analyses were done by StW and SaW. The analysis and interpretation of the results was carried out by IDB, StW, SaW, EO, MvdB, and SL. IDB, StW, and SL drafted the manuscript, and all authors read and approved the final manuscript.

## Conflict of Interest Statement

The authors declare that the research was conducted in the absence of any commercial or financial relationships that could be construed as a potential conflict of interest.

## Footnotes

1 www.IedereenWetenschapper.be

## References

[B1] Arumugam M. Raes J. Pelletier E. Le Paslier D. Yamada T. Mende D. R. (2011). Enterotypes of the human gut microbiome. *Nature* 473 174–180. 10.1038/nature09944 21508958 PMC3728647

[B2] Bassis C. M. Tang A. L. Young V. B. Pynnonen M. A. (2014). The nasal cavity microbiota of healthy adults. *Microbiome* 2:27. 10.1186/2049-2618-2-27 25143824 PMC4138944

[B3] Biesbroek G. Sanders E. A. M. Roeselers G. Wang X. Caspers M. P. M. Trzciñski K. (2012). Deep sequencing analyses of low density microbial communities: working at the boundary of accurate microbiota detection. *PLOS ONE* 7:e32942. 10.1371/journal.pone.0032942 22412957 PMC3295791

[B4] Biesbroek G. Tsivtsivadze E. Sanders E. A. M. Montijn R. Veenhoven R. H. Keijser B. J. F. (2014). Early respiratory microbiota composition determines bacterial succession patterns and respiratory health in children. *Am. J. Respir. Crit. Care Med.* 190 1283–1292. 10.1164/rccm.201407-1240OC 25329446

[B5] Biswas K. Hoggard M. Jain R. Taylor M. W. Douglas R. G. (2015). The nasal microbiota in health and disease: variation within and between subjects. *Front. Microbiol.* 9:134. 10.3389/fmicb.2015.00134 25784909 PMC4345913

[B6] Bogaert D. Keijser B. Huse S. Rossen J. Veenhoven R. van Gils E. (2011). Variability and diversity of nasopharyngeal microbiota in children: a metagenomic analysis. *PLOS ONE* 6:e17035. 10.1371/journal.pone.0017035 21386965 PMC3046172

[B7] Bosch A. A. de Steenhuijsen Piters W. A. van Houten M. A. Chu M. L. J. N. Biesbroek G. Kool J. (2017). Maturation of the infant respiratory microbiota, environmental drivers and health consequences: a prospective cohort study. *Am. J. Respir. Crit. Care Med.* 10.1164/rccm.201703-0554OC [Epub ahead of print]. 28665684

[B8] Callahan B. J. McMurdie P. J. Holmes S. P. (2017). Exact sequence variants should replace operational taxonomic units in marker-gene data analysis. *ISME J.* 10.1038/ismej.2017.119 [Epub ahead of print]. 28731476 PMC5702726

[B9] Callahan B. J. Mcmurdie P. J. Rosen M. J. Han A. W. Johnson A. J. Holmes S. P. (2016). DADA2: high-resolution sample inference from Illumina amplicon data. *Nat. Methods* 13 581–583. 10.1038/nMeth.3869 27214047 PMC4927377

[B10] Camarinha-Silva A. Jáuregui R. Chaves-Moreno D. Oxley A. P. A. Schaumburg F. Becker K. (2014). Comparing the anterior nare bacterial community of two discrete human populations using Illumina amplicon sequencing. *Environ. Microbiol.* 16 2939–2952. 10.1111/1462-2920.12362 24354520

[B11] Caporaso J. G. Lauber C. L. Walters W. A. Berg-lyons D. Lozupone C. A. Turnbaugh P. J. (2010). Global patterns of 16S rRNA diversity at a depth of millions of sequences per sample. *Proc. Natl. Acad. Sci. U.S.A.* 108(Suppl. 1) 4516–4522. 10.1073/pnas.1000080107 20534432 PMC3063599

[B12] Charlson E. S. Chen J. Custers-Allen R. Bittinger K. Li H. Sinha R. (2010). Disordered microbial communities in the upper respiratory tract of cigarette smokers. *PLOS ONE* 5:e15216. 10.1371/journal.pone.0015216 21188149 PMC3004851

[B13] Chonmaitree T. Jennings K. Golovko G. Khanipov K. Pimenova M. Patel J. A. (2017). Nasopharyngeal microbiota in infants and changes during viral upper respiratory tract infection and acute otitis media. *PLOS ONE* 12:e0180630. 10.1371/journal.pone.0180630 28708872 PMC5510840

[B14] Cremers A. J. Zomer A. L. Gritzfeld J. F. Ferwerda G. van Hijum S. A. Ferreira D. M. (2014). The adult nasopharyngeal microbiome as a determinant of pneumococcal acquisition. *Microbiome* 2:44. 10.1186/2049-2618-2-44 25671106 PMC4323220

[B15] de Steenhuijsen Piters W. A. A. Sanders E. A. M. Bogaert D. (2015). The role of the local microbial ecosystem in respiratory health and disease. *Philos. Trans. R. Soc. B Biol. Sci.* 370:20140294. 10.1098/rstb.2014.0294 26150660 PMC4528492

[B16] de Vries S. P. W. Bootsma H. J. Hays J. P. Hermans P. W. M. (2009). Molecular aspects of *Moraxella catarrhalis* pathogenesis. *Microbiol. Mol. Biol. Rev.* 73 389–406. 10.1128/MMBR.00007-09 19721084 PMC2738133

[B17] Duell B. L. Su Y. C. Riesbeck K. (2016). Host–pathogen interactions of nontypeable *Haemophilus influenzae*: from commensal to pathogen. *FEBS Lett.* 590 3840–3853. 10.1002/1873-3468.12351 27508518

[B18] Eren A. M. Morrison H. G. Lescault P. J. Reveillaud J. Vineis J. H. Sogin M. L. (2014). Minimum entropy decomposition: unsupervised oligotyping for sensitive partitioning of high-throughput marker gene sequences. *ISME J.* 9 968–979. 10.1038/ismej.2014.195 25325381 PMC4817710

[B19] Eren A. M. Sogin M. L. Maignien L. (2016). Editorial: new insights into microbial ecology through subtle nucleotide variation. *Front. Microbiol.* 7:1318 10.3389/fmicb.2016.01318 27605925 PMC4995221

[B20] Falcony G. Joossens M. Vieira-Silva S. Wang J. Darzi Y. Faust K. (2016). Population-level analysis of gut microbiome variation. *Science* 352 560–564. 10.1126/science.aad3503 27126039

[B21] Francis N. A. Butler C. C. Hood K. Simpson S. Wood F. Nuttall J. (2009). Effect of using an interactive booklet about childhood respiratory tract infections in primary care consultations on reconsulting and antibiotic prescribing: a cluster randomised controlled trial. *BMJ* 339:b2885. 10.1136/bmj.b2885 19640941 PMC2718088

[B22] Goldstein E. J. C. Murphy T. F. Parameswaran G. I. (2009). *Moraxella catarrhalis*, a human respiratory tract pathogen. *Clin. Infect. Dis.* 49 124–131. 10.1086/599375 19480579

[B23] Hugerth L. W. Andersson A. F. (2017). Analysing microbial community composition through amplicon sequencing: from sampling to hypothesis testing. *Front. Microbiol.* 8:1561. 10.3389/fmicb.2017.01561 28928718 PMC5591341

[B24] Koren O. Knights D. Gonzalez A. Waldron L. Segata N. Knight R. (2013). A guide to enterotypes across the human body: meta-analysis of microbial community structures in human microbiome datasets. *PLOS Comput. Biol.* 9:e1002863. 10.1371/journal.pcbi.1002863 23326225 PMC3542080

[B25] Kozich J. J. Westcott S. L. Baxter N. T. Highlander S. K. Schloss P. D. (2013). Development of a dual-index sequencing strategy and curation pipeline for analyzing amplicon sequence data on the MiSeq Illumina sequencing platform. *Appl. Environ. Microbiol.* 79 5112–5120. 10.1128/AEM.01043-13 23793624 PMC3753973

[B26] Laufer A. S. Metlay J. P. Gent J. F. Fennie K. P. Kong Y. Pettigrew M. M. (2011). Microbial communities of the upper respiratory tract and otitis media in children. *mBio* 2:e00245-10. 10.1128/mBio.00245-10 21285435 PMC3031303

[B27] Lax S. Smith D. P. Hampton-Marcell J. Owens S. M. Handley K. M. Scott N. M. (2014). Longitudinal analysis of microbial interaction between humans and the indoor environment. *Science* 345 1048–1052. 10.1126/science.1254529 25170151 PMC4337996

[B28] Lemon K. (2010). Comparative analyses of the bacterial microbiota of the human nostril and oropharynx. *mBio* 1:e00129-10. 10.1128/mBio.00129-10 20802827 PMC2925076

[B29] Ling Z. Liu X. Luo Y. Yuan L. Nelson K. E. Wang Y. (2013). Pyrosequencing analysis of the human microbiota of healthy Chinese undergraduates. *BMC Genomics* 14:390. 10.1186/1471-2164-14-390 23758874 PMC3685588

[B30] Man W. H. de Steenhuijsen Piters W. A. A. Bogaert D. (2017). The microbiota of the respiratory tract: gatekeeper to respiratory health. *Nat. Rev. Microbiol.* 15 259–270. 10.1038/nrmicro.2017.14 28316330 PMC7097736

[B31] Oksanen J. Blanchet F. Kindt R. Legendre P. O’Hara R. (2016). *Vegan: Community Ecology Package. R Package 2.3-3.* 10.4135/9781412971874.n145

[B32] Petrova M. I. Lievens E. Malik S. Imholz N. Lebeer S. (2015). *Lactobacillus* species as biomarkers and agents that can promote various aspects of vaginal health. *Front. Physiol.* 6:81. 10.3389/fphys.2015.00081 25859220 PMC4373506

[B33] Roos K. Håkansson E. G. Holm S. (2001). Effect of recolonisation with “interfering” α streptococci on recurrences of acute and secretory otitis media in children: randomised placebo controlled trial. *BMJ* 322 210–212. 10.1136/bmj.322.7280.210 11159619 PMC26587

[B34] Santee C. A. Nagalingam N. A. Faruqi A. A. DeMuri G. P. Gern J. E. Wald E. R. (2016). Nasopharyngeal microbiota composition of children is related to the frequency of upper respiratory infection and acute sinusitis. *Microbiome* 4 34. 10.1186/s40168-016-0179-9 27364497 PMC4929776

[B35] Stearns J. C. Davidson C. J. McKeon S. Whelan F. J. Fontes M. E. Schryvers A. B. (2015). Culture and molecular-based profiles show shifts in bacterial communities of the upper respiratory tract that occur with age. *ISME J.* 9 1246–1259. 10.1038/ismej.2014.250 25575312 PMC4409167

[B36] Tano K. Grahn Håkansson E. Holm S. E. Hellström S. (2002). A nasal spray with alpha-haemolytic streptococci as long term prophylaxis against recurrent otitis media. *Int. J. Pediatr. Otorhinolaryngol.* 62 17–23. 10.1016/S0165-5876(01)00581-X 11738689

[B37] Teo S. M. Mok D. Pham K. Kusel M. Serralha M. Troy N. (2015). The infant nasopharyngeal microbiome impacts severity of lower respiratory infection and risk of asthma development. *Cell Host Microbe* 17 704–715. 10.1016/j.chom.2015.03.008 25865368 PMC4433433

[B38] Tibshirani R. Walther G. (2005). Cluster validation by prediction strength. *J. Comput. Graph. Stat.* 14 511–528. 10.1198/106186005X59243

[B39] Turnbaugh P. J. Ley R. E. Hamady M. Fraser-Liggett C. M. Knight R. Gordon J. I. (2007). The human microbiome project. *Nature* 449 804–810. 10.1038/nature06244 17943116 PMC3709439

[B40] van der Poll T. Opal S. M. (2009). Pathogenesis, treatment, and prevention of pneumococcal pneumonia. *Lancet* 374 1543–1556. 10.1016/S0140-6736(09)61114-4 19880020

[B41] van der Velden A. Duerden M. Bell J. Oxford J. Altiner A. Kozlov R. (2013). Prescriber and patient responsibilities in treatment of acute respiratory tract infections — essential for conservation of antibiotics. *Antibiotics* 2 316–327. 10.3390/antibiotics2020316

[B42] Wickham H. (2009). *ggplot2: Elegant Graphics for Data Analysis.* New York, NY: Springer.

[B43] Yu G. Phillips S. Gail M. H. Goedert J. J. Humphrys M. S. Ravel J. (2017). The effect of cigarette smoking on the oral and nasal microbiota. *Microbiome* 5 3. 10.1186/s40168-016-0226-6 28095925 PMC5240432

