# Supplementary material for: Comparing the Healthy Nose and Nasopharynx Microbiota Reveals Continuity As Well As Niche-Specificity
Source: Front Microbiol. 2017 Nov 29;8:2372. doi: 10.3389/fmicb.2017.02372 (PMC5712567; doi:10.3389/fmicb.2017.02372)
Supplement: Supplementary file 1 [file Data_Sheet_1.docx]

# **Supplementary Material_Tables**

**Table S1: Evolution of total library size after sequencing.** Number of reads are presented after different filtering steps. Of note, these are read counts for all samples analyzed on the same sequencing run (n = 246), of which 176 samples were used in our analysis.

| Filtering step | Amount of reads |
| --- | --- |
| sequencing | 9212351 |
| removing_low_quality | 5882456 |
| removing_unmergable | 4360521 |
| removing_chimeras | 4328922 |
| removing_too_long | 4325664 |
| removing_non_bacterial | 4232836 |
| removing_pcr_contaminants | 4183119 |
| removing_extraction_contaminants | 4140489 |

**Table S2: Frequency rates of the tested covariates in this study.** For the allergy data, categories ‘FALSE’ indicate non-allergic participants, categories ‘TRUE’ indicate allergic participants. For the smoking data, category ‘FALSE’ indicates non-smokers, whereas category ‘TRUE’ indicates smokers and ex-smokers. NA = not available. All covariates were tested for possible associations with the nasopharyngeal bacterial profiles.

| Variable | category | number |
| --- | --- | --- |
| gender | M | 34 |
| gender | F | 58 |
| age | 18-45 | 77 |
| age | 45-65 | 15 |
| smoker | FALSE | 76 |
| smoker | TRUE | 16 |
| blood_type | A- | 9 |
| blood_type | A+ | 18 |
| blood_type | AB- | 2 |
| Blood_typ | AB+ | 1 |
| blood_type | B- | 2 |
| blood_type | B+ | 2 |
| blood_type | O- | 6 |
| blood_type | O+ | 31 |
| Blood_type | NA | 21 |
| season | autumn | 22 |
| season | spring | 19 |
| season | winter | 22 |
| season | summer | 29 |
| IgE_total | FALSE | 71 |
| IgE_total | TRUE | 13 |
| IgE_total | NA | 8 |
| sIgE_house_dust_mite | FALSE | 64 |
| sIgE_house_dust_mite | TRUE | 20 |
| sIgE_house_dust_mite | NA | 8 |
| sIgE_grass_pollen | FALSE | 64 |
| sIgE_grass_pollen | TRUE | 20 |
| sIgE_grass_pollen | NA | 8 |
| sIgE_tree_pollen | FALSE | 72 |
| sIgE_tree_pollen | TRUE | 12 |
| sIgE_tree_pollen | NA | 8 |

# **Supplementary Material_Figures**

**
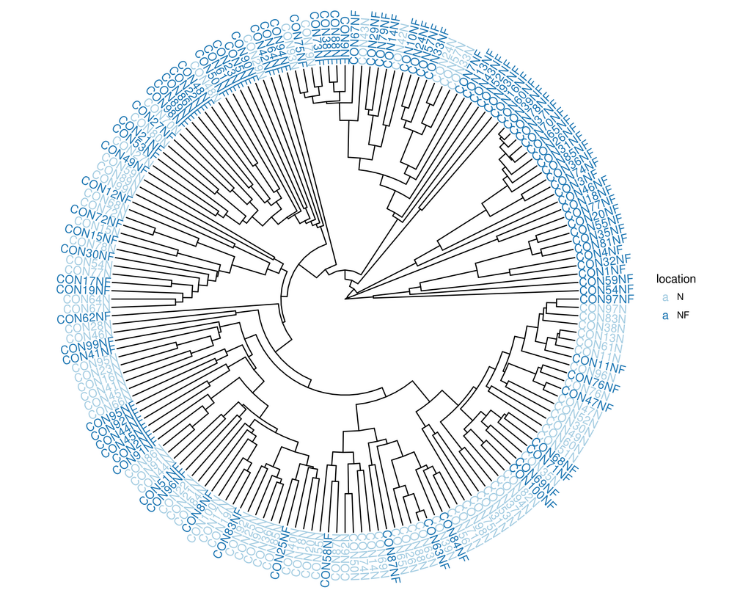
**

**Figure S1: Dendrogram of hierarchical clustering of nose and nasopharynx samples.**

**
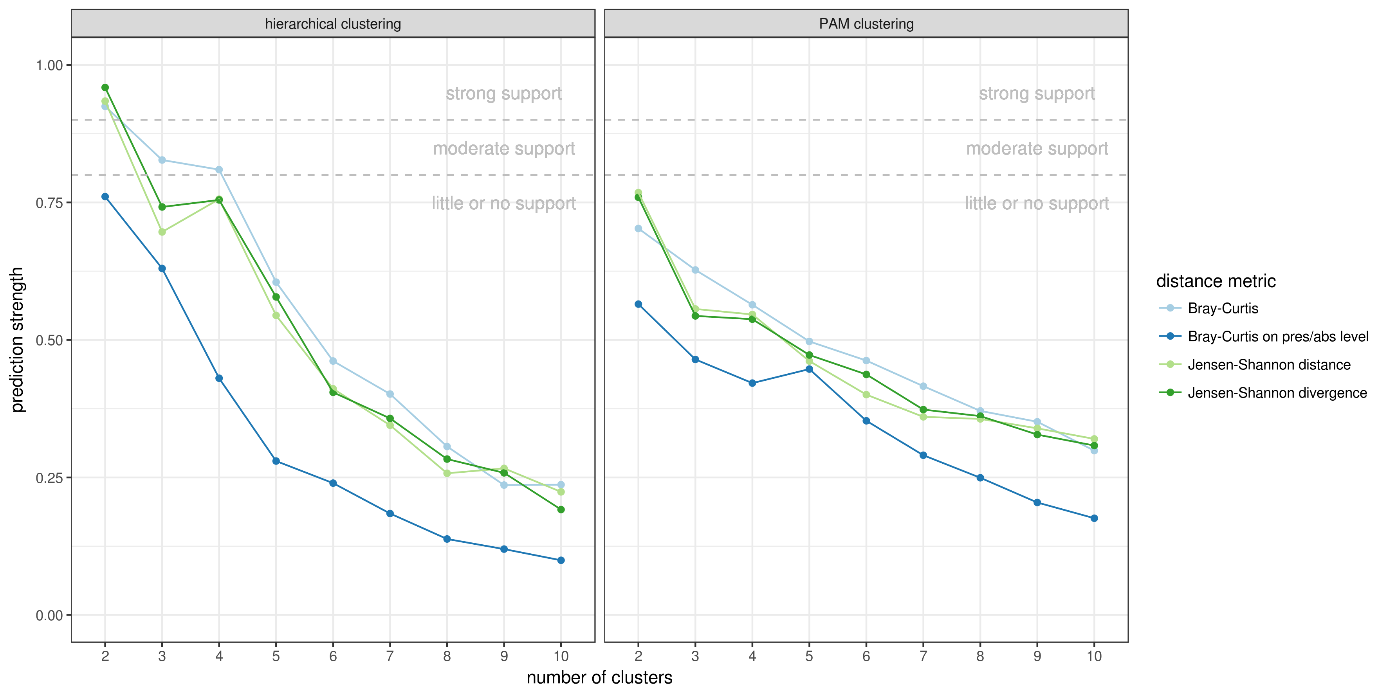
**

**Figure S2: Prediction strength to confirm presence of nasal and nasopharyngeal bacterial community types for both hierarchical (left) and PAM clustering (right).** Prediction strength was measured based on four distance matrices: Bray-Curtis (on relative abundances, as usual), Bray-Curtis on the presence/absence level, Jensen-Shannon divergence and Jensen-Shannon distance (equal to the square root of the Jensen-Shannon divergence). For hierarchical clustering, strong to moderate support was observed for significance for up to four clusters. For PAM clustering, little or no support was observed for significant clusters in our dataset.

**
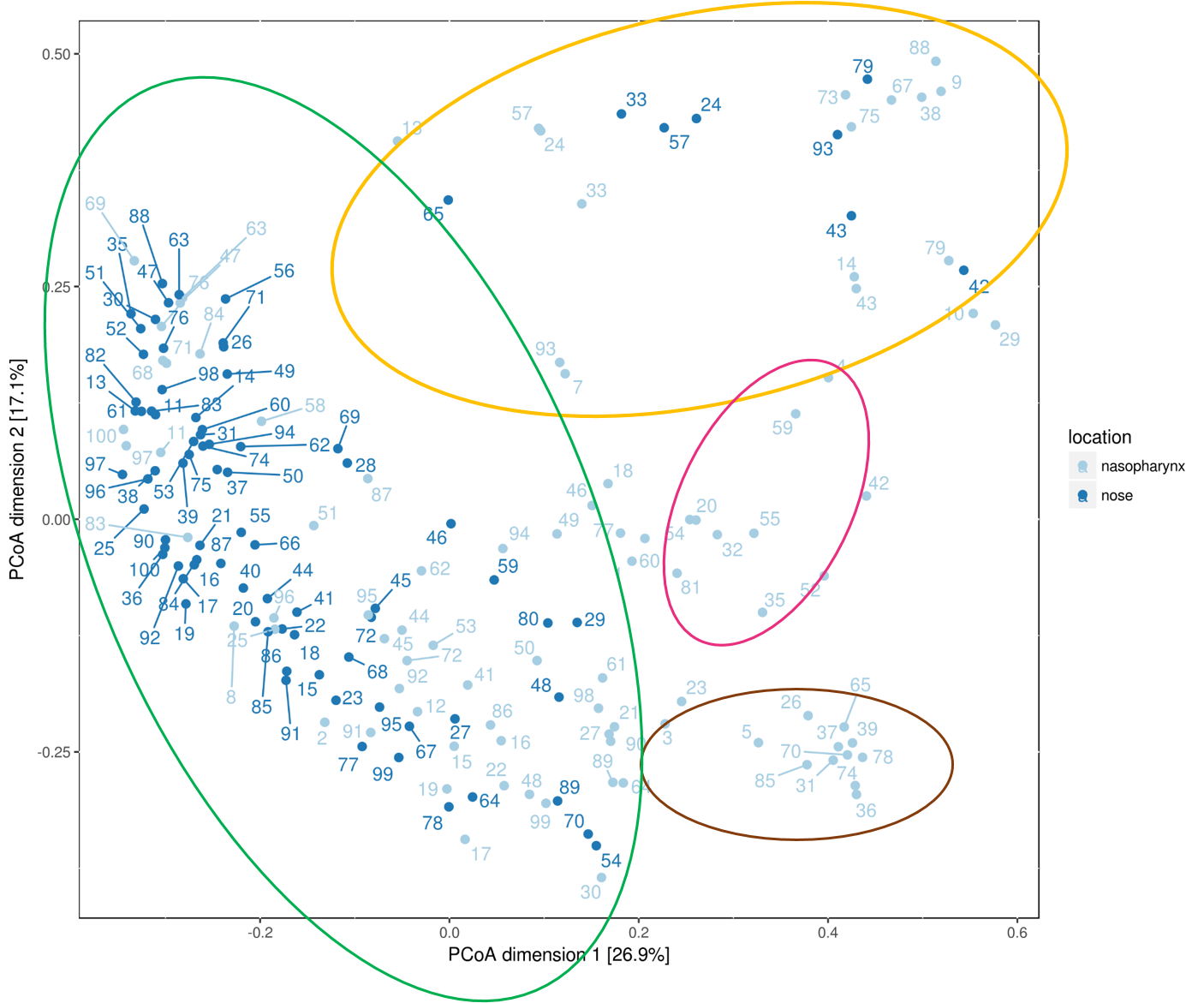
**

**Figure S3: Principal Coordinate Analysis (PCoA) with visualization of the different observed bacterial ‘community types’.** The bacterial ‘community types’ that were observed upon hierarchical clustering are visualized in this PCoA. Orange = *Moraxella*-dominated, brown = *Streptococcus*-dominated, pink = *Fusobacterium*-dominated and green = intermixed profile with key members *Staphylococcus*, *Dolosigranulum* and *Corynebacterium*. Also three smaller clusters were seen after hierarchical clustering. Since prediction strength evaluation only supported significance for up to four clusters, we believe that the significance of the three smaller clusters (*Neisseria*, *Alloprevotella* and *Haemophilus*) should be confirmed in larger study groups.

.


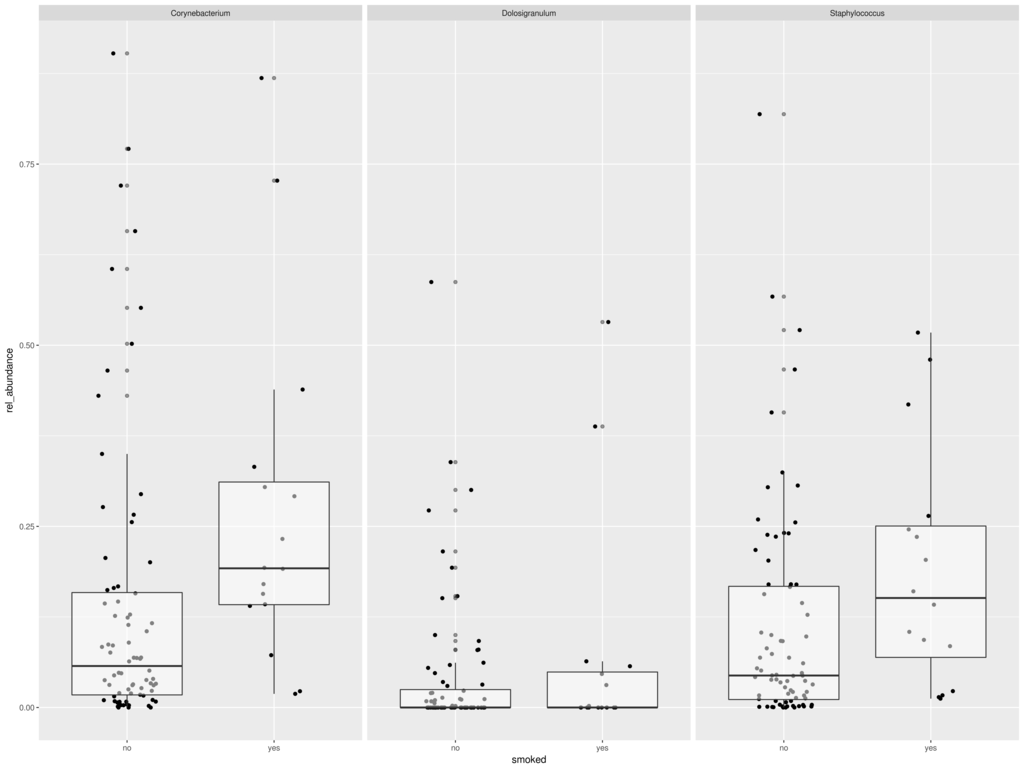


**Figure S4: Correlation between smoking and the genera *Corynebacterium*, *Staphylococcus* and *Dolosigranulum*.** A positive association was found at genus level between smoking and *Corynebacterium* (p = 0.002) and *Staphylococcus* (p = 0.02).


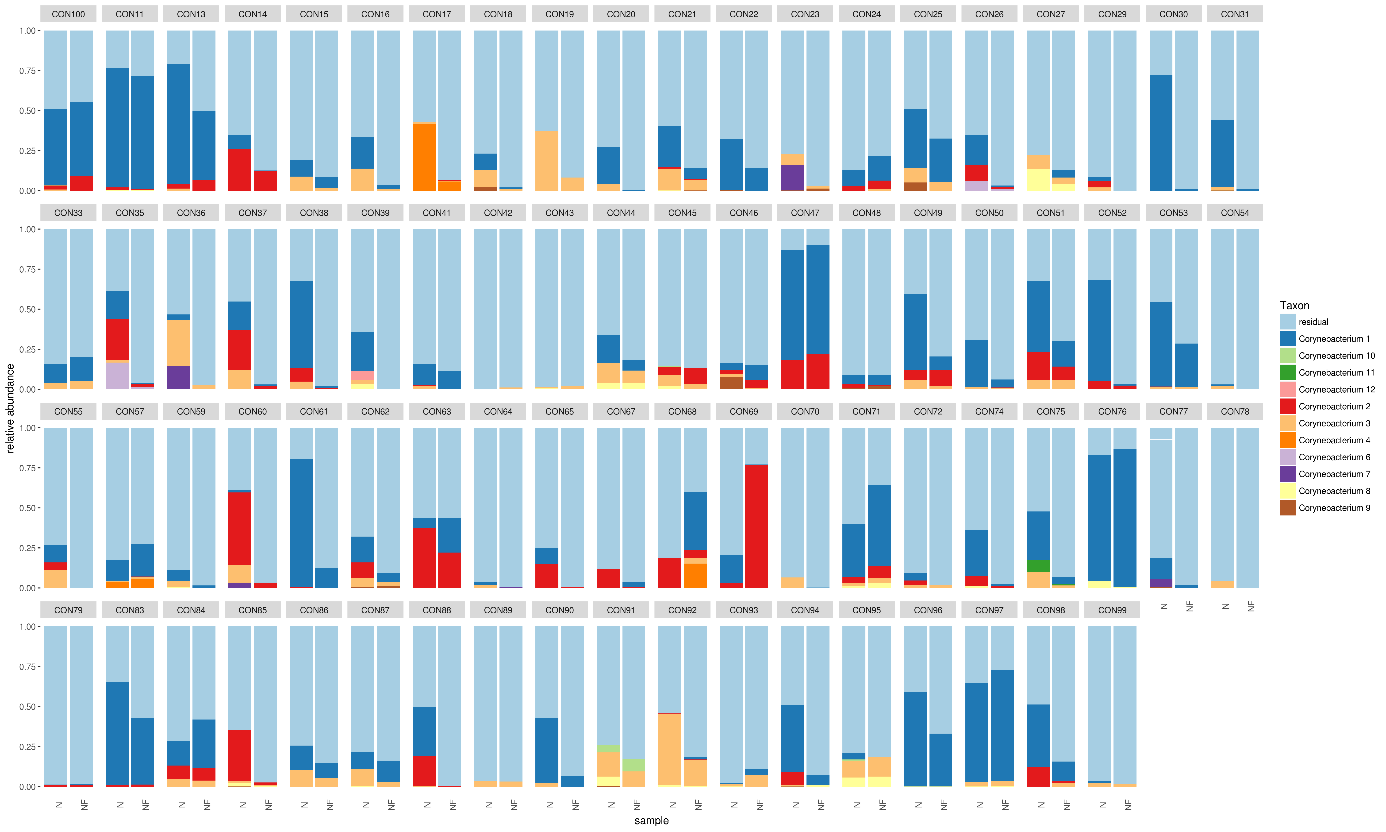


**Figure S5a**: Relative abundance of different *Corynebacterium* ASVs in paired nose and nasopharynx samples.


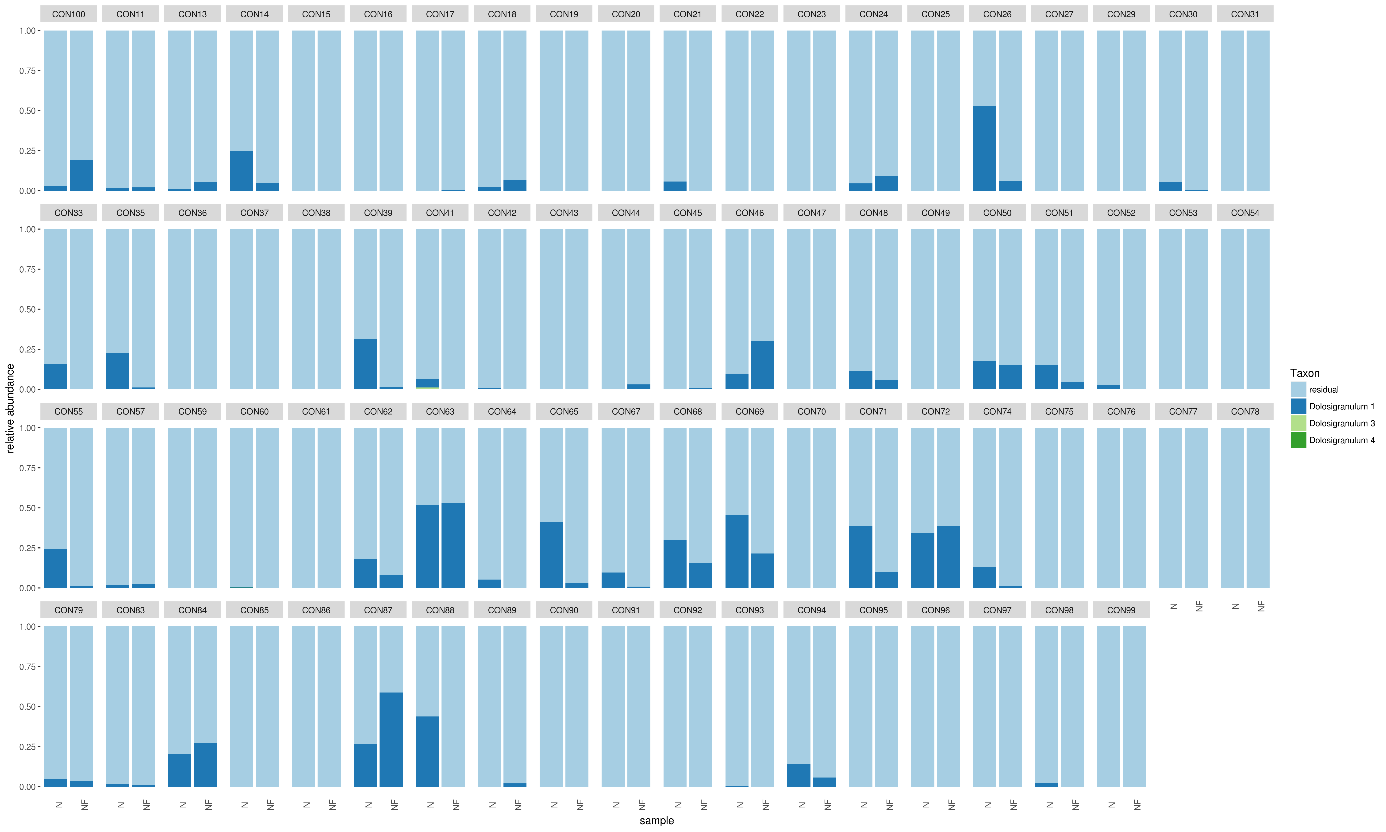


**Figure S5b**: Relative abundance of *Dolosigranulum* ASVs in paired nose and nasopharynx samples.


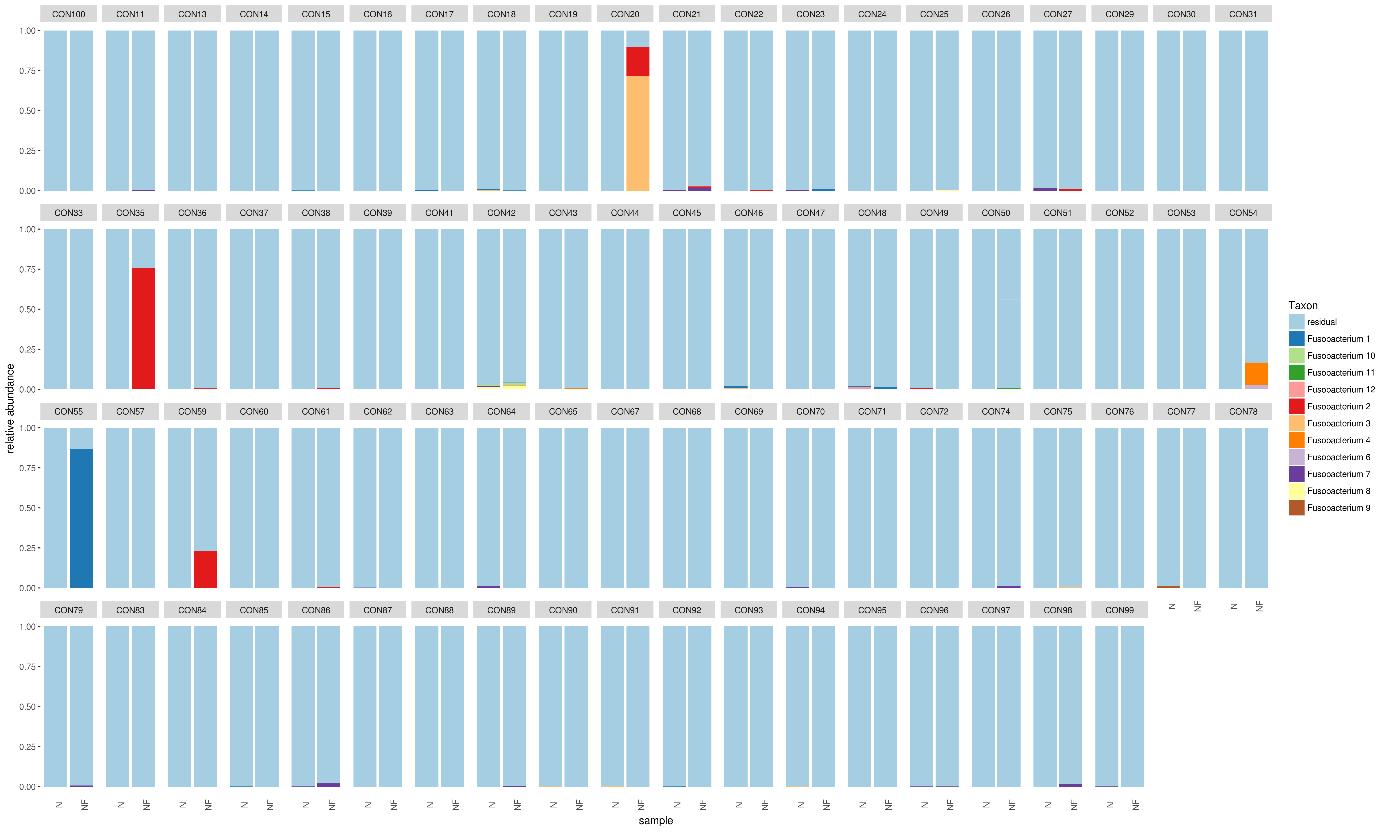


**Figure S5c**: Relative abundance of different *Fusobacterium* ASVs in paired nose and nasopharynx samples.


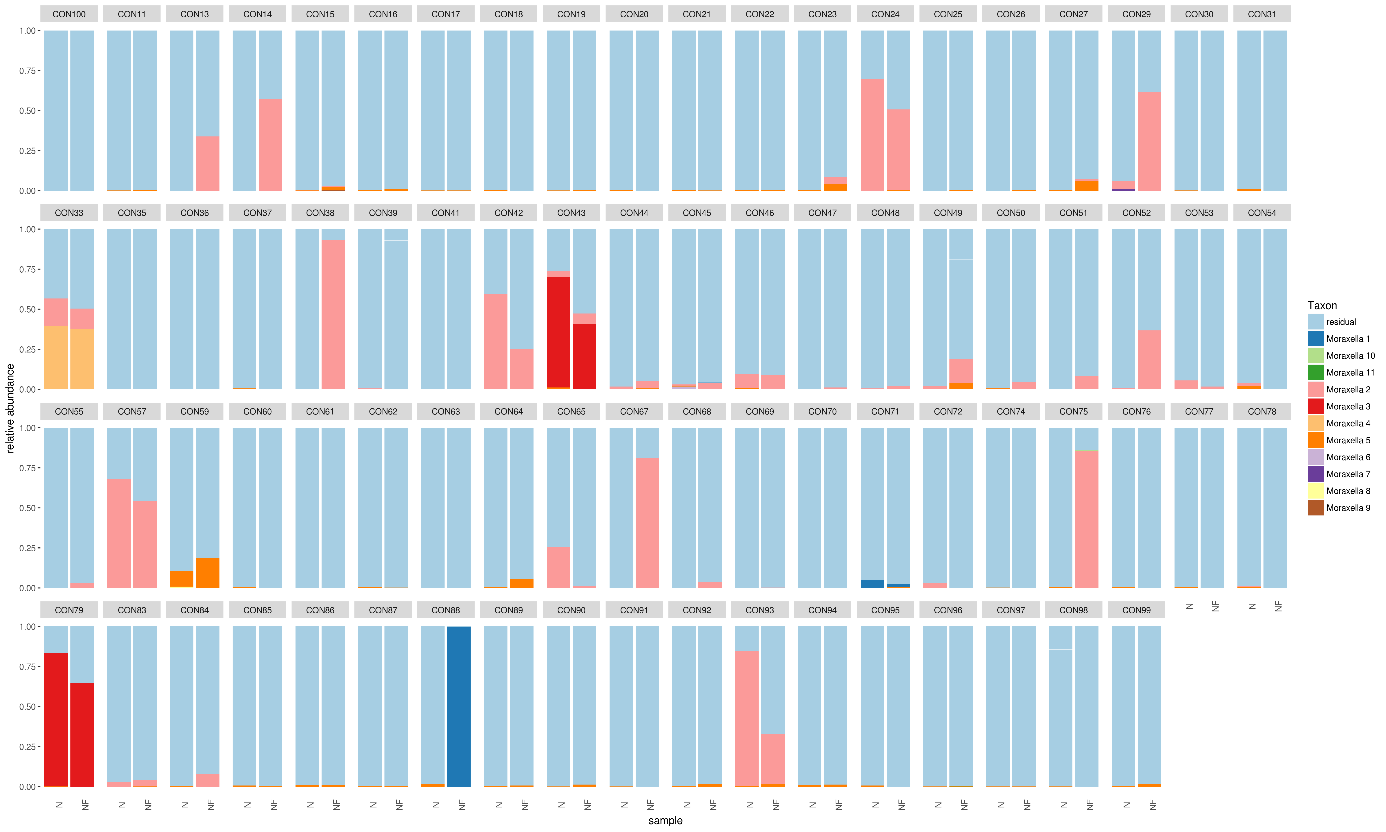


**Figure S5d**: Relative abundance of different *Moraxella* ASVs in paired nose and nasopharynx samples.


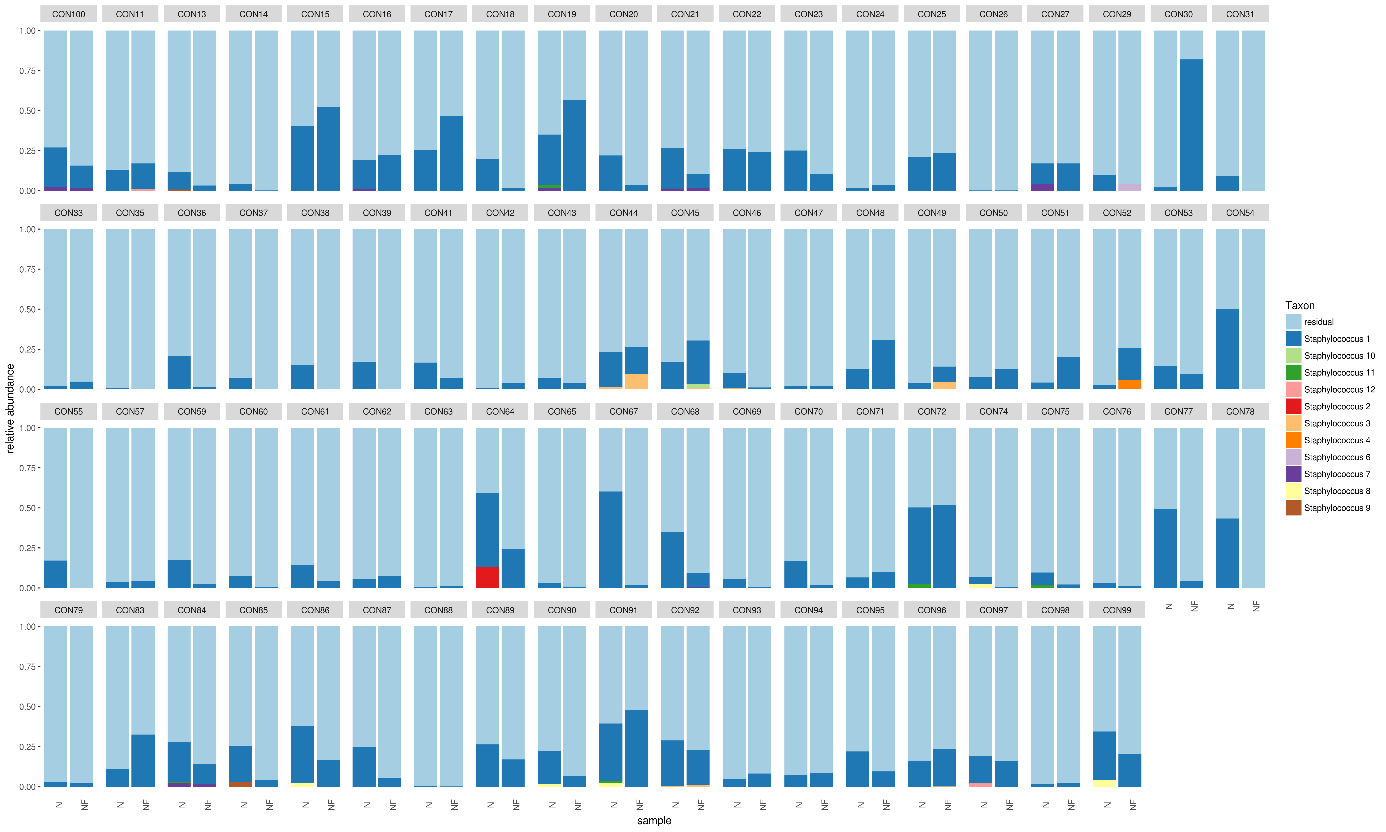


**Figure S5e**: Relative abundance of different *Staphylococcus* ASVs in paired nose and nasopharynx samples.


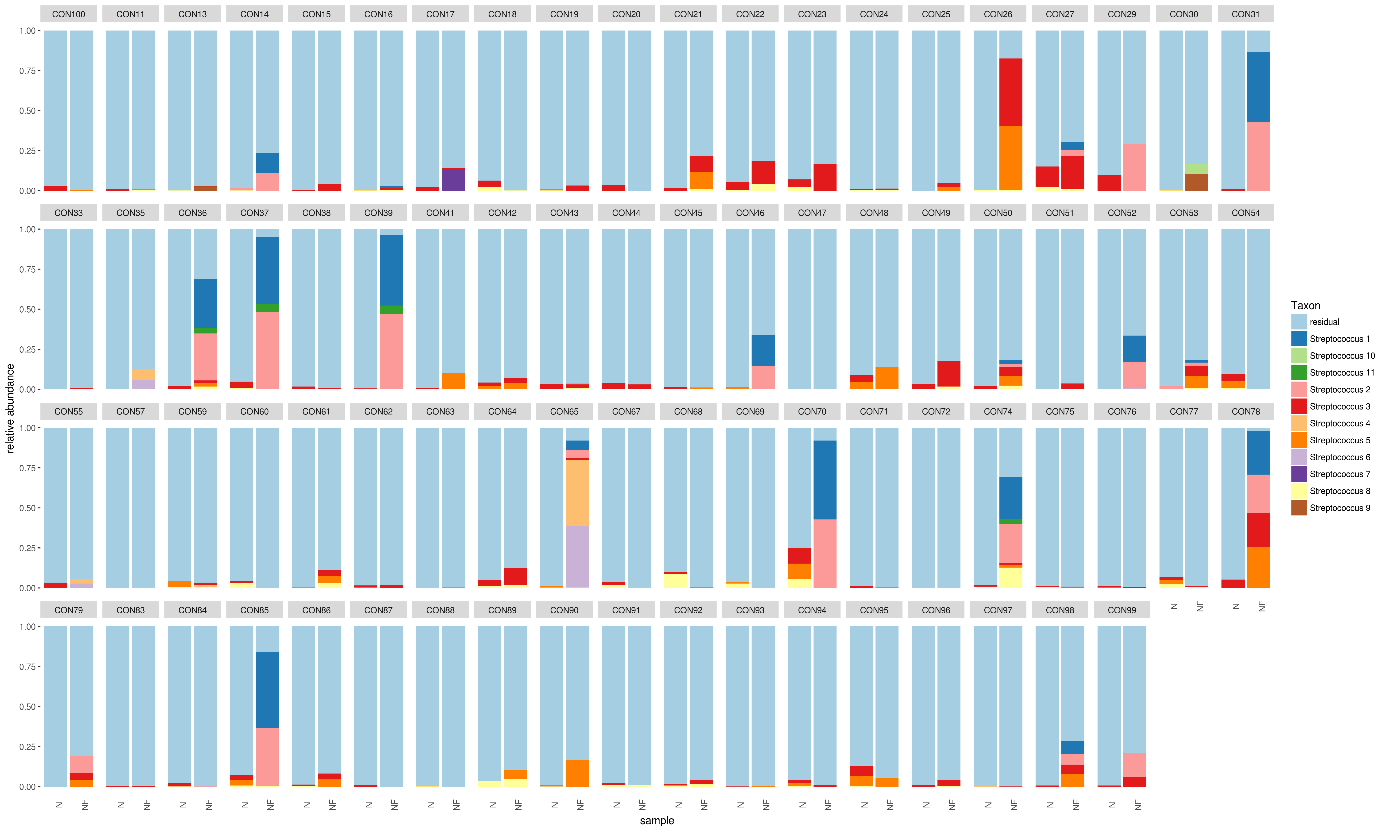


**Figure S5f**: Relative abundance of different *Streptococcus* ASVs in paired nose and nasopharynx samples.


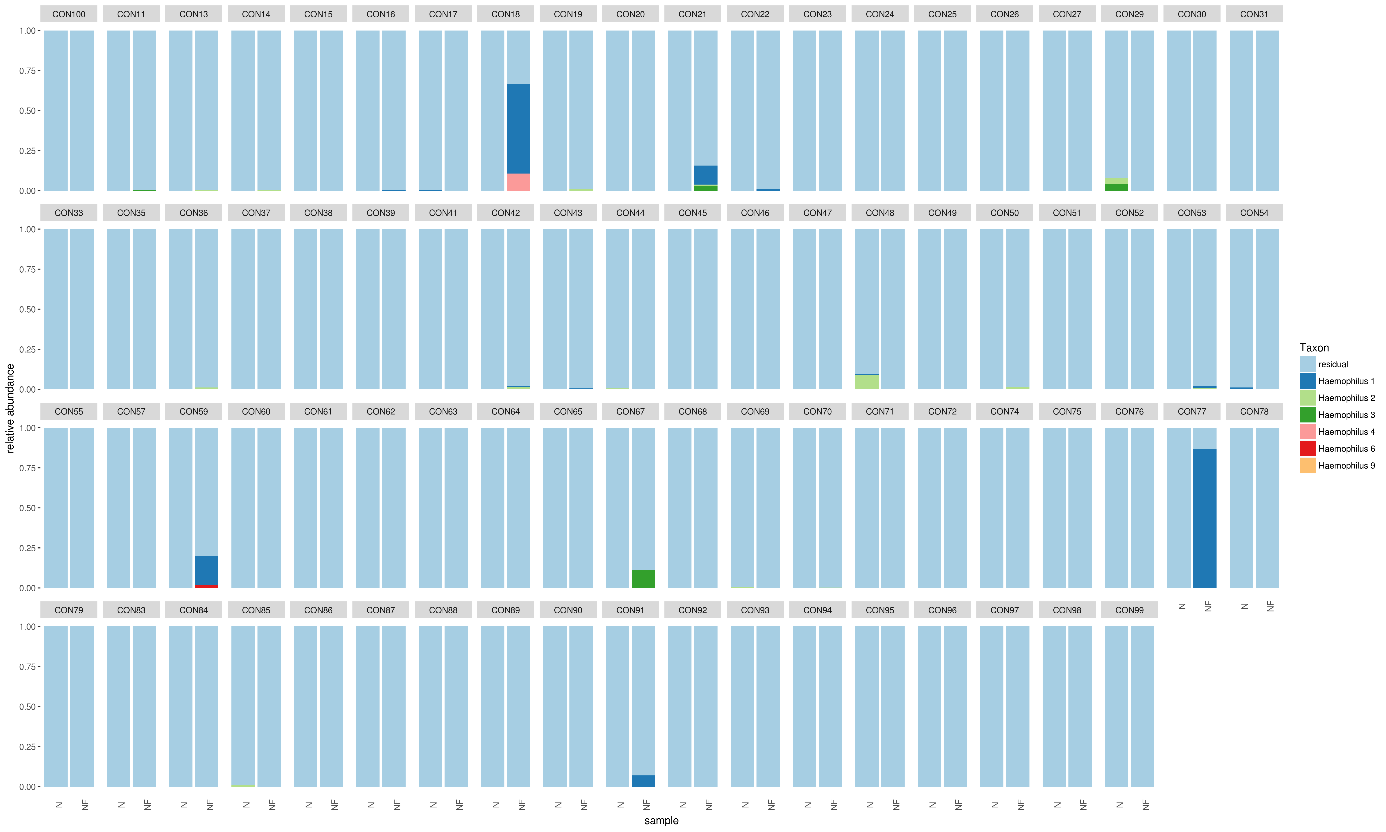


**Figure S5g**: Relative abundance of different *Haemophilus* ASVs in paired nose and nasopharynx samples.


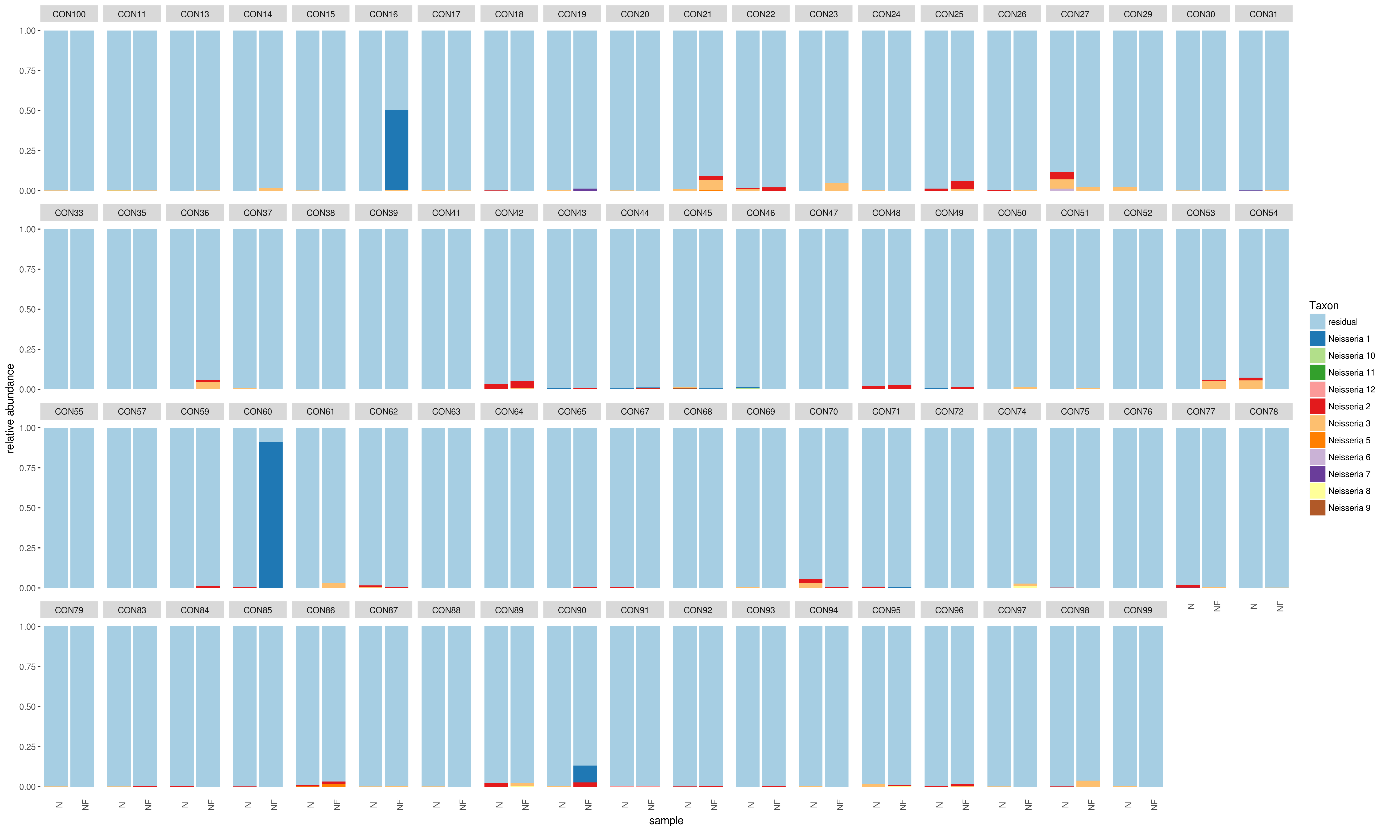


**Figure S5h**: Relative abundance of different *Neisseria* ASVs in paired nose and nasopharynx samples.

**
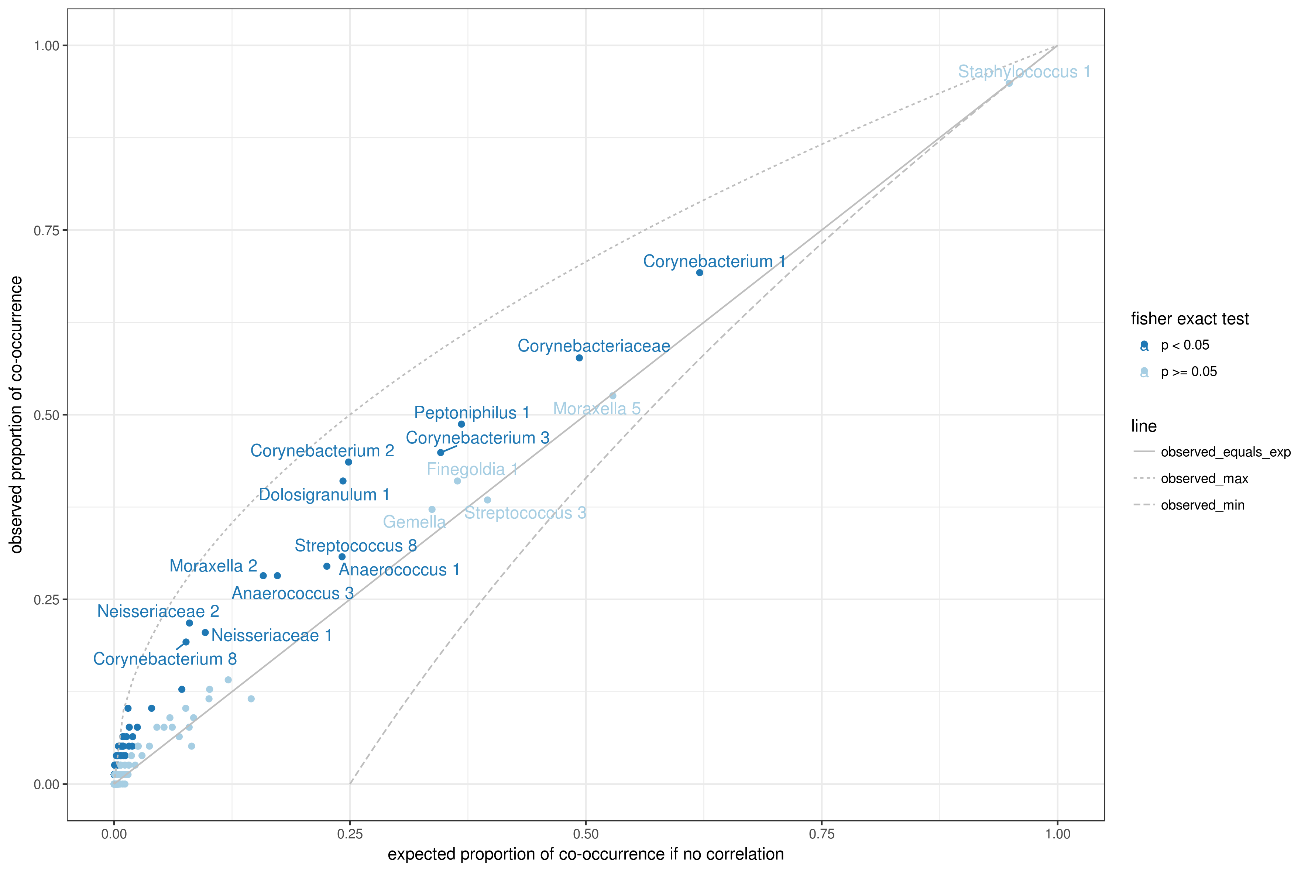
**

**Figure S6:** Correlation between presence of ASVs in the nose and the nasopharynx. A Fisher exact test was used to test significance of ASVs that show co-occurrence (p < 0.05).


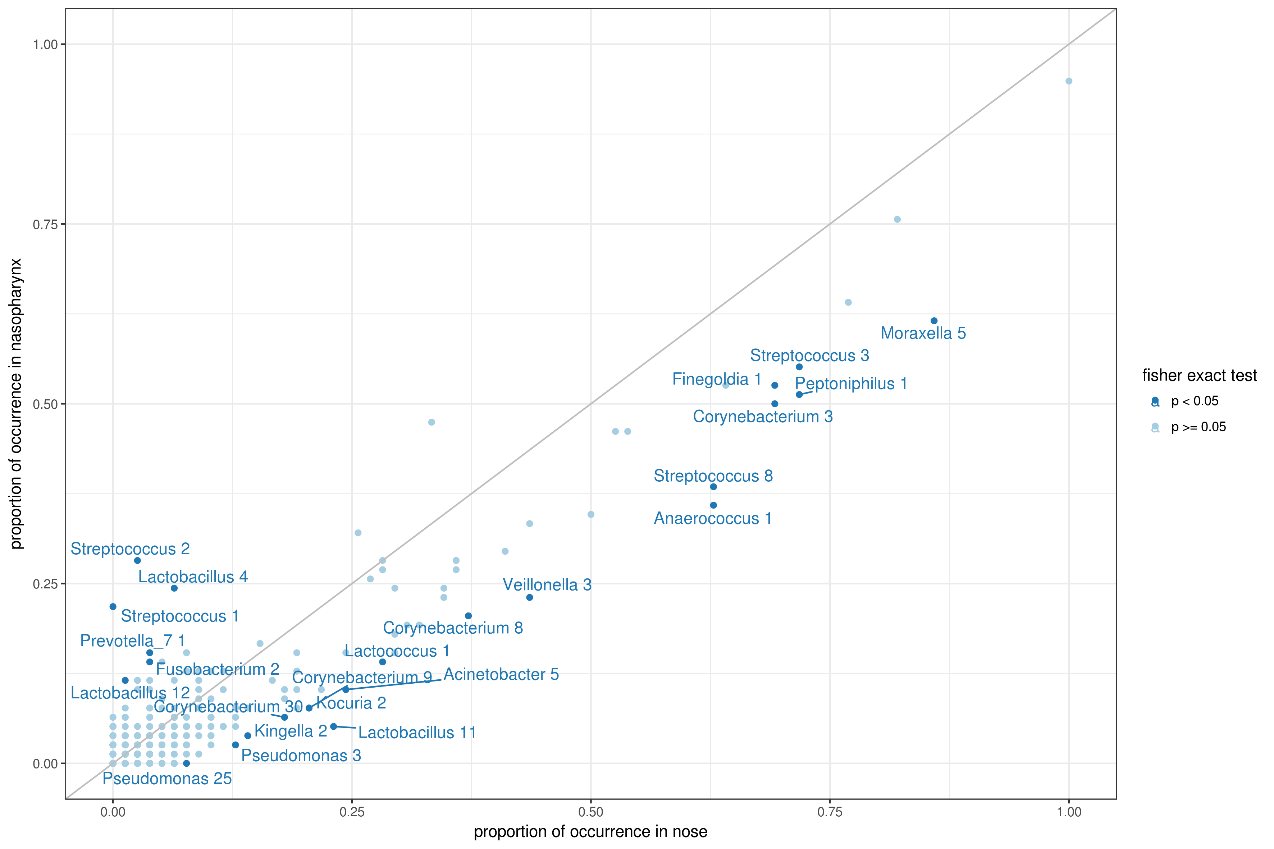


**Figure S7:** Presence of ASVs in the nose and the nasopharynx to study niche-specificity. A Fisher exact test was used to test significance of ASVs that show niche-specificity (p < 0.05).
